# Iron Induces Anti-tumor Activity in Tumor-Associated Macrophages

**DOI:** 10.3389/fimmu.2017.01479

**Published:** 2017-11-08

**Authors:** Milene Costa da Silva, Michael O. Breckwoldt, Francesca Vinchi, Margareta P. Correia, Ana Stojanovic, Carl Maximilian Thielmann, Michael Meister, Thomas Muley, Arne Warth, Michael Platten, Matthias W. Hentze, Adelheid Cerwenka, Martina U. Muckenthaler

**Affiliations:** ^1^Department of Pediatric Oncology, Hematology and Immunology, University of Heidelberg, Heidelberg, Germany; ^2^Molecular Medicine Partnership Unit (MMPU), Heidelberg University, European Molecular Biology Laboratory (EMBL), Heidelberg, Germany; ^3^Graduate Program in Areas of Basic and Applied Biology (GABBA), Abel Salazar Biomedical Sciences Institute (ICBAS), University of Porto, Porto, Portugal; ^4^Innate Immunity Group, German Cancer Research Center (DKFZ), Heidelberg, Germany; ^5^Translational Lung Research Center Heidelberg (TLRC), German Center for Lung Research (DZL), University of Heidelberg, Heidelberg, Germany; ^6^Department of Neuroradiology, University Hospital Heidelberg, Heidelberg, Germany; ^7^German Cancer Consortium, Clinical Cooperation Unit Neuroimmunology and Brain Tumor Immunology, German Cancer Research Center (DKFZ), Heidelberg, Germany; ^8^Translational Research Unit, Thoraxklinik at University Hospital Heidelberg, Heidelberg, Germany; ^9^Institute of Pathology, University of Heidelberg, Heidelberg, Germany; ^10^Division of Immunbiochemistry, Medical Faculty Mannheim, Heidelberg University, Heidelberg, Germany

**Keywords:** tumor-associated macrophages, macrophage polarization, hemolytic red blood cells, heme, iron, non-small cell lung cancer, iron nanoparticles, anti-tumor activity

## Abstract

Tumor-associated macrophages (TAMs) frequently help to sustain tumor growth and mediate immune suppression in the tumor microenvironment (TME). Here, we identified a subset of iron-loaded, pro-inflammatory TAMs localized in hemorrhagic areas of the TME. The occurrence of iron-loaded TAMs (iTAMs) correlated with reduced tumor size in patients with non-small cell lung cancer. *Ex vivo* experiments established that TAMs exposed to hemolytic red blood cells (RBCs) were converted into pro-inflammatory macrophages capable of directly killing tumor cells. This anti-tumor effect could also be elicited *via* iron oxide nanoparticles. When tested *in vivo*, tumors injected with such iron oxide nanoparticles led to significantly smaller tumor sizes compared to controls. These results identify hemolytic RBCs and iron as novel players in the TME that repolarize TAMs to exert direct anti-tumor effector function. Thus, the delivery of iron to TAMs emerges as a simple adjuvant therapeutic strategy to promote anti-cancer immune responses.

## Introduction

The tumor microenvironment (TME) significantly influences tumor progression (1). It is characterized by high cellular complexity, including fibroblasts, stroma, and blood vessels, and infiltrates of immune cells. In several human cancers, tumor-associated macrophages (TAMs) are a major immune component of the TME (2–4). In particular, non-small cell lung cancer (NSCLC) was shown to have one of the highest TAMs densities when compared to other cancers, such as liver, ovary, breast, and prostate cancer (5). In general, macrophages display a high degree of functional plasticity, reflected by their capacity to integrate diverse signals from the microenvironment and to acquire distinct phenotypes (6–11).

In the TME, pro-inflammatory “M1 macrophages” counteract tumor growth either by activating adaptive immune responses or by directly killing tumor cells (12–15). By contrast, anti-inflammatory “M2 macrophages” sustain tumor cell growth (16) by promoting angiogenesis, matrix remodeling, and immune suppression (17, 18). Undesirably, most TAMs display the M2-like phenotype, sustaining tumor growth rather than supporting tumor elimination. Thus, reprogramming macrophages in the TME could represent a promising therapeutic strategy to improve anti-tumor activity (19).

Besides contributing to immune responses, macrophages play a critical role in the recycling of iron from red blood cells (RBCs). Macrophages located in the spleen and the liver engulf senescent RBCs and catabolize heme via the activity of heme oxygenases (HO-1 and HO-2) (20). Iron is either stored in ferritin or exported *via* ferroportin, the only known iron exporter (21, 22).

Recently, we discovered that anti-inflammatory macrophages shift toward the pro-inflammatory state after exposure to heme or iron (23). Additional studies supported the concept that iron can drive macrophages toward a pro-inflammatory phenotype (24, 25). These findings interconnect the dual functions of macrophages in iron handling and inflammation.

M1 macrophages are hallmarked by the production of reactive oxygen species (ROS) and pro-inflammatory cytokines, such as interleukin (IL)-1α/β, IL-6, tumor necrosis factor alpha (TNFα), and also by expression of inducible nitric oxide synthase (iNOS), cluster of differentiation (CD)86, major histocompatibility complex II, and CD14 (26, 27). They retain iron as a result of high levels of ferritin and low ferroportin expression (28, 29). By contrast, M2 macrophages produce anti-inflammatory cytokines, such as IL-10 and transforming growth factor beta (TGFβ), and are hallmarked by the expression of arginase 1, Ym1, and CD206 (26, 27). They express more ferroportin and less ferritin compared to M1 macrophages and display an “iron-recycling” phenotype (28, 29).

So far, the consequences of macrophage exposure to hemolytic RBCs were studied in hemolytic disease (e.g., sickle cell disease), where M1-like reprogramming by heme and iron aggravates tissue damage (23). We now explore the responses of TAMs to neoangiogenesis, which nourishes the tumor but also causes extravasation of RBCs and the release of heme and iron. We demonstrate that hemolysis in the TME reprograms TAMs to a pro-inflammatory phenotype, which shows an important role in inducing anti-tumor activity. Iron oxide nanoparticles can mimic these responses, suggesting a therapeutic strategy that can be exploited for (immunotherapy-based) anti-cancer approaches.

## Materials and Methods

### NSCLC Paraffin Slides

Paraffin slides were provided by the Lung Biobank Heidelberg, a member of the Biomaterial bank Heidelberg (BMBH) and the Biobank platform of the German Center for Lung Research (DZL). Tissue microarrays (TMAs) were provided by the tissue bank of the National Center for Tumor Diseases (NCT, Heidelberg, Germany), in accordance with the regulations of the tissue bank and the approval of the ethics committee of Heidelberg University. TMA classification was performed according to the sixth edition of the Tumor Node Metastasis staging system for NSCLC. Paraffin slides from 19 patients with NSCLC (Table 1) were analyzed under the microscope and divided into iron positive and iron negative according to the visible detection/absence of intracellular iron. Each paraffin slide included tumor center, invasive front and tumor periphery.

**Table 1:** Clinicopathological characterization of patients (*n* = 19) from histology slides of non-small cell lung cancer.

Variable	Iron positive (*n* = 11)	Iron negative (*n* = 8)
Age (years, mean ± SEM)	64.00 ± 2.676	55.13 ± 3.777
Gender (male:female)	9:2	7:1
Survival [live:dead (%)]	4:7 (57)	1:7 (14)
Histology: number (%)		
Adenocarcinoma	4 (36)	4 (50)
Squamous	6 (55)	4 (50)
Large cell	1 (9)	0
Tumor grade: number (%)		
1	1 (9)	0
2	4 (36)	4 (50)
3	6 (55)	4 (50)
Presence of RBCs near iron positive cells (number of positive:negative slides)	10:1	n.a.
Smoker: number (%)	11 (100)	8 (100)

*n.a., not applicable*.

### Tumor Suspensions

Human adenocarcinoma tumors were obtained from NSCLC patients (*n* = 4) who underwent resection for primary lung cancer at the Thoraxklinik of the University Hospital, Heidelberg, Germany. Fresh tumors (human and mouse) were mechanically dissociated and digested with DNAse (SIGMA) and Hyaluronidase (SIGMA). Tumor suspensions were strained using a 70 µm cell strainer (Becton Dickinson) and washed with PBS. For the isolation of human leukocytes, cell suspensions were layered over a density gradient solution (Biocoll Separating Solution, 1.077 g/ml, Biochrom AG, Germany) in a 1:1 volume ratio (450 g, 30 min, RT). Leukocytes were collected, washed twice (PBS) and resuspended (PBS, 4°C). Cells were further processed for magnetic isolation. For the preparation of mouse tumors, a gradient purification using a Lympholyte solution (Cederlane) was performed to remove RBCs and dead cells. Briefly, 7 ml of tumor suspension were added on top of 7 ml of Lympholyte solution and centrifuged (1,500 × *g*, 25 min, 20°C). The layer of live cells was removed and washed again in cold PBS. The cell pellet was resuspended in PBS (4°C) and kept on ice for the respective procedures.

### Magnetic Isolation

Iron-loaded macrophages were isolated from cell suspensions as described in Ref. (30). Briefly, cell suspensions were resuspended in 5 ml of PBS and passed through an LS column attached to a magnetic board (Miltenyi Biotech). Columns were washed three times with 5 ml of PBS. Cells that were adherent to the column (magnetic fraction) were flushed with 5 ml PBS. Cells were either resuspended in PBS and centrifuged for cytospin preparations or lysed for RNA extraction.

### Tumor Model

Female and male [C57BL/6N and Slc40a1^C326S/C326S^ (31)] mice were used at 8- to 10-weeks of age. Experiments were approved by “Regierungspräsidium Karlsruhe” under the project number G267/12. Lewis lung carcinoma (LLC) cells were injected (1 × 10^6^ in 100 µl PBS) subcutaneously into the flanks of mice. When indicated, LLC cells were co-injected with cross-linked iron oxide (CLIO)-FITC nanoparticles (8 mg of iron/kg of mouse), or only in PBS. Tumor size was assessed by caliper measurements at the indicated time points and volumes were calculated using the following formula: V = 1/2(length [mm] × width [mm]^2^) as previously described (32). Mice were sacrificed at the indicated time points. Mice that developed ulcers or necrotic tumors were sacrificed and not considered for the experiments. Blood was removed directly from the heart by cardiac puncture. Subcutaneous tumors were resected and dissected carefully to avoid tissue damage and bleedings induced during animal preparation. Tumors were transferred to PBS on ice and tumor weight (g) was measured on a scale. Tumors were processed for cytospin, FACS analysis and FACS sorting, snap frozen until further analysis or fixed in formalin for immunohistochemistry and histological analysis.

### Dissection of Hemorrhagic Areas from LLC Tumors

After careful resection, LLC tumors were washed in PBS and placed in a petry dish under a stereo microscope (Olympus SZ51). Tumors were evaluated for the presence of hemorrhagic areas. Hemorrhagic (H) areas were distinguished from non-hemorrhagic (NH) areas by a strong red coloration. With the help of tweezers and scalpel, H and NH areas from the same tumor were dissected, separated, and stored accordingly for further processing. As validation and quality control for the dissection procedure, heme was quantified.

### Histology and Immunohistology

Single cell suspensions (200 µl) were centrifuged (500 r.p.m, 5 min) in a Cytospin Cytocentrifuge (Thermo Scientific). Tissues were fixed for 24 h in 10% neutral buffered formalin (Sigma-Aldrich), dehydrated, and embedded in paraffin. Tissue sections (3–5 µm) were stained for iron using Accustain Iron Stain No. HT20 (Sigma-Aldrich) following manufacturer’s instructions. When indicated, Perls’ blue staining was further enhanced using the DAB peroxidase substrate kit SK-4100 (Vector Labs). Quantification of iron staining was performed using the Image Pro-Premier 3D software. The software calculated the area of pixels corresponding to blue staining (iron staining). For immunostaining, cytospin samples were fixed and permeabilized in ice-cold acetone for 5 min, washed in PBS and treated with H_2_O_2_ to block endogenous peroxidase. For immunohistochemistry, sections were treated for 10 min with 3% H_2_O_2_ (Sigma-Aldrich) and subjected to microwave-mediated antigen retrieval using the Citraplus reagent (Biogenex). Immunostaining was performed according to the instructions of the Vectastain ABC mouse, rat, and rabbit kits (Vector Labs). Anti-mouse ferroportin staining was performed using MTP11-A rabbit polyclonal antibody (Anti-Mouse Metal Transporter Protein1/Ferroportin (MTP1/IREG1/Fpn) from Alpha Diagnostics); rabbit IgG was used as isotype control for the ferroportin staining. Anti-human CD68 staining was performed using the monoclonal mouse anti-human PG-M1 clone (DAKO). Tissue slides were developed using the Vector AEC substrate (Vector Labs), rinsed with distilled water, counterstained with hematoxylin, washed in PBS, and mounted using the VectaMount AQ mounting medium (Vector Labs). Images were acquired with a Ni-E Nikon microscope.

### Preparation of *In Vitro* TAMs

Bone marrow cells were flushed from tibia and femur using ice-cold HBSS and filtered through a 70 µm cell strainer. Cells were seeded at a density of 700,000 cells/ml in equal volumes of conditioned media (CM) from LLC cells (or DMEM used as control) and complete RPMI1640-Glutamax medium (Life Technologies) [supplemented with 10% of heat-inactivated FBS (Thermo Scientific), 1% penicillin/streptomycin (Sigma-Aldrich) and 10 ng/ml M-CSF (Sigma-Aldrich)]. For 12 well plates, cells were incubated with 0.5 ml of CM plus 0.5 ml of complete RPMI. For 6 well plates, cells were incubated with 1 ml of CM plus 1 ml of complete RPMI. After 4 days, the medium was removed and centrifuged (1,500 r.p.m for 5 min) to remove cells in suspension. Macrophages were incubated in the same media with additional CM for 24 h (0.5 ml for 12 well plates and 1 ml for 6 well plates). For each independent experiment, BMDM were prepared from three different mice. At least three independent experiments were performed for each figure panel.

### RBC Preparation

Red blood cell aging was performed as described (33). Mouse blood was collected on EDTA tubes, washed with PBS (3×), and resuspended in Hepes buffer (10 mM Hepes, 140 mM NaCl, BSA 0.1%, pH 7.4). Cells kept at 4°C overnight were considered non-aged RBCs. For *in vitro* RBCs aging (aRBC), cells (1 × 10^8^ cells/ml in Hepes buffer) were incubated overnight at 30°C with 2.5 mM calcium and 0.5 mM of the ionophore A23187 (Calbiochem). Before incubation with TAMs, both RBCs fractions were washed twice (PBS, 1,500 r.p.m, 5 min) and resuspended in RPMI.

### Statistical Analysis

Data are shown as mean ± SEM, and the number of mice (*n*) is indicated. Statistical analyses were performed using Prism v.6 (GraphPad). Comparisons between two groups were performed with two-sided Welch *t*-tests, and among three or more than three groups with one-way ANOVA, followed by Bonferroni post-test. *p < 0.05, **p < 0.01, ***p < 0.001, and ****p < 0.0001 are indicated.

## Results

### Iron Accumulates in a Subset of TAMs in Human NSCLC

**Figure 1:**
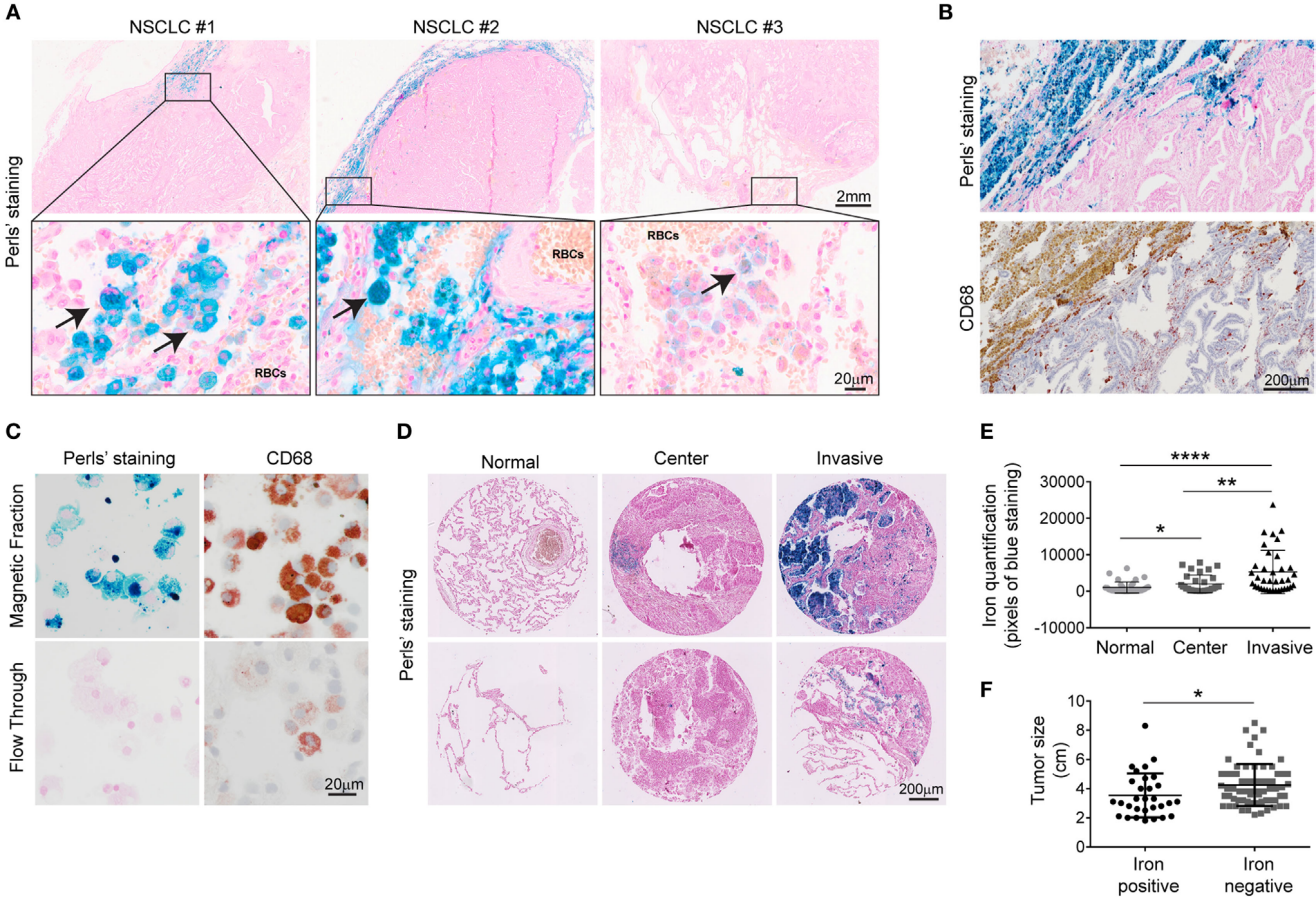
Tumor-associated macrophages associated with invasive margin accumulate iron and correlate with smaller tumor size. **(A)** Representative examples of three different patients with non-small cell lung cancer (NSCLC). Arrows indicate iron-positive cells (blue staining). Red blood cells (RBCs) are identified by morphology. **(B,C)** Representative examples of Perls’ staining and anti-CD68 immunostaining in lung adenocarcinoma **(B)** and tumor-associated leukocytes after magnetic isolation **(C)**, blue staining indicates iron and red staining represents CD68 positive cells (representative of four patients). **(D)** Representative Perls’ staining in normal lung, tumor center and invasive front in lung squamous cell carcinoma (upper panel) and lung adenocarcinoma (lower panel). **(E)** Quantification of Perls’ staining in normal lung, center and invasion front of NSCLC. Results are shown as area of pixels corresponding to blue staining (*n* = 38). **(F)** Comparison of tumor size in a cohort of NSCLC patients divided by iron content: iron positive (*n* = 29) and iron negative (*n* = 65). Data are shown as mean ± SEM. *p < 0.05, **p < 0.01, ***p < 0.001, and ****p < 0.0001.

To explore whether iron is detectable in the TME, Perls’ staining was performed on human NSCLC pathological specimen (*n* = 19 samples). Iron positive staining was detected in 11 cases while 8 samples were iron negative. Table 1 summarizes the available clinical information from these patients grouped by the iron staining results. While cancer cells were negative for iron staining in all samples, some infiltrating cells were clearly iron positive. Interestingly, iron positive cells accumulated in the vicinity of RBCs (10/11 patients) (Figure 1A; Table 1). In addition, iron staining strongly overlapped with positive immune staining for CD68, a macrophage marker (Figure 1B). To further analyze if iron accumulates in TAMs, we isolated leukocytes from fresh tumor tissue (human lung adenocarcinoma) (Table 2) and separated them by exposure to a magnetic field according to their iron content. Cells retained in the magnetic fraction (due to their high iron content) stained positive for iron while cells in the flow through were negative for iron (Figure 1C). Cells within the magnetic fraction were strongly CD68 positive demonstrating that TAMs accumulate iron in lung adenocarcinoma. In addition the macrophage population in the flow through was iron negative (Figure 1C). To explore whether iron-loaded TAMs (iTAMs) are associated with specific areas in the tumor, we performed iron staining in TMA from 116 patients with NSCLC. From each patient, three areas of the original histology block were represented: normal lung, tumor center, and the invasion front (Figure 1D). 38 of 116 patient samples stained positive for iron in the tumor center and/or the invasion front (Table 3). Quantification of iron staining revealed that signals are significantly higher in the invasion front and tumor center when compared to normal lung (Figure 1E). We next analyzed if iron content correlates with tumor size (length in centimeters). Even if it represents a relatively crude clinical parameter, the tumor size of patients scored as iron positive was significantly smaller compared to iron negative tumors (Figure 1F) independently of the histological subtype (Figure S1A in Supplementary Material) and tumor grade (Figure S1B in Supplementary Material). Taken together these data demonstrate that iron accumulates in a subset of TAMs that localize in the vicinity of RBCs in invasive areas of the tumor. Furthermore, the presence of iTAMs correlates with smaller tumor size.

**Table 2:** Clinicopathological characterization of patients (*n* = 4) of non-small cell lung carcinoma (fresh tumors).

	Patient			
Variable	1^a^	2	3	4
Age	73	54	74	69
Gender	Female	Female	Male	Male
Histology	Adenocarcinoma	Adenocarcinoma	Adenocarcinoma	Adenocarcinoma
Tumor grade	2	3	2	2

*^a^Cytospin slides from this patient are shown in Figure 1C*.

**Table 3:** Clinicopathological characterization of patients (*n* = 116) from tissue microarrays (TMAs) of non-small cell lung carcinoma.

Variable	Iron positive (*n* = 38)	Iron negative (*n* = 78)	*p*-Value
Age (years, mean ± SEM)	61.65 ± 1.364	64.82 ± 0.8501	0.0453 (*)
Gender (male:female)	28:10	61:17	
Histology: number (%)			
Adenocarcinoma	18 (47)	28 (36)	
Squamous	20 (53)	45 (58)	
Large cell	0	5(6)	
Tumor grade: number (%)			
1	0	1 (1)	
2	10 (26)	30 (38)	
3	28 (74)	47 (66)	
Survival [live:dead (%)]	17:21 (80)	29:49 (60)	

*p < 0.05.

### iTAMs Show Increased Expression of Markers for Iron Import and Decreased Expression of the Iron Export Protein Ferroportin

**Figure 2:**
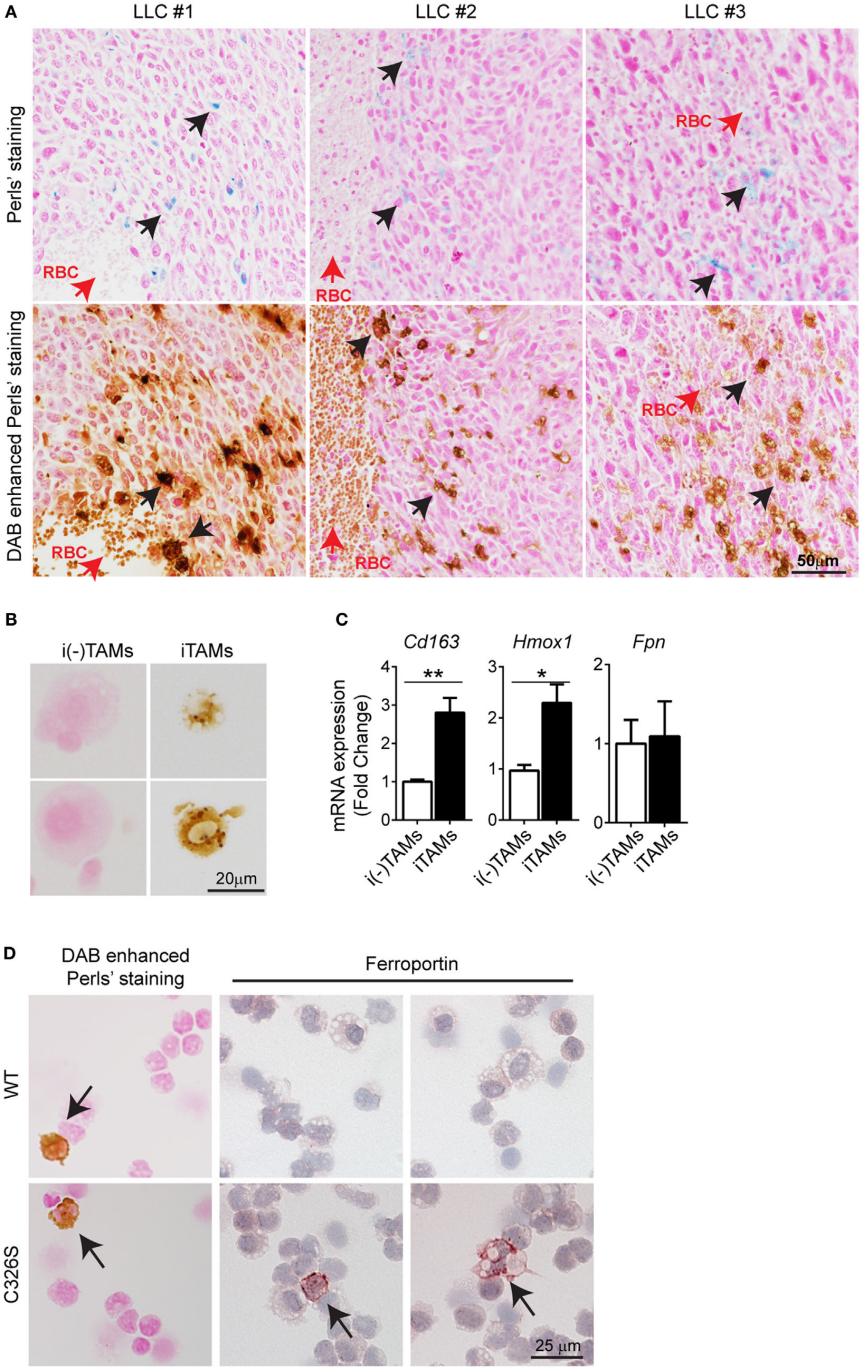
Iron-loaded tumor-associated macrophages (TAMs) from Lewis lung carcinoma (LLC) tumors localize near sites of red blood cells (RBCs) extravasation. **(A)** Consecutive slides with Perls’ staining of three different LLC tumors (upper panel) and DAB enhanced Perls’ staining (lower panel). Black arrows indicate iron-loaded TAMs and red arrows indicate RBCs. **(B)** DAB enhanced Perls’ staining of sorted iron-spared (i(-)TAMs) and iron-loaded (iTAMs) TAMs after magnetic isolation. **(C)** mRNA expression of *Cd163, Hmox1*, and *Fpn* in iron-spared (i(-)TAMs) and iron-loaded (iTAMs) TAMs determined by quantitative RT-PCR (three independent experiments, each experiment with TAMs pooled from eight mice). **(D)** DAB enhanced Perls’ staining and anti-ferroportin staining in TAMs sorted from LLC tumors of WT and Slc40a1^C326S^ mice (C326S). Images are representative of 4 mice and arrows indicate TAMs positive for iron and ferroportin staining respectively. All mRNA levels were normalized to *Rpl19* mRNA expression and all tissues were collected 15 days after LLC inoculation. Data are shown as mean ± SEM. *p < 0.05, **p < 0.01, ***p < 0.001, and ****p < 0.0001.

To understand the molecular mechanism(s) of how iron accumulates in TAMs, we used the LLC mouse model, a widely used syngeneic model for NSCLC (34). Consistent with our findings in human NSCLC, infiltrating cells in the proximity of RBCs stained positive for iron (Figure 2A), whereas cancer cells were negative for iron staining. We next isolated TAMs from LLC tumors and sorted for the surface markers CD11b^+^/Gr-1^−^/F4/80^+^ (35) by flow cytometry (see gating strategy in Figure S2A in Supplementary Material). We further separated them into iron-spared (i(-)TAMs) and iron-loaded TAMs (iTAMs) by magnetic isolation (Figure 2B). iTAMs express elevated mRNA levels of *Cd163* [the scavenger receptor for hemoglobin and haptoglobin-hemoglobin complexes (36), and *Hmox1* (the inducible isoform of heme oxygenase responsible for heme degradation)], while mRNA expression of ferroportin (*Fpn*), was low and similar to iron-spared TAMs (Figure 2C). At the protein level, ferroportin was not detectable in TAMS, but in splenic macrophages that were analyzed as a control (Figure S3A in Supplementary Material). Ferroportin is internalized and degraded by the binding of the hepatic iron-hormone hepcidin. Inflammation increases hepcidin levels and decreases ferroportin cell surface expression causing iron retention in macrophages (37–42). Tumor-bearing mice neither showed elevated serum levels of the inflammatory cytokines IL-6 and IL-1β, known activators of hepcidin expression (43, 44) (Figure S3B in Supplementary Material) nor increased hepatic hepcidin mRNA levels (*Hamp1*) (Figure S3C in Supplementary Material). Consistently, the expression of ferroportin was detectable in cell types contributing to systemic iron supplies, such as splenic macrophages, Kupffer cells and enterocytes (Figure S3D in Supplementary Material). Taken together these data suggest that iron retention in TAMs does not depend on a hepcidin-dependent decrease of ferroportin expression. Consistently, TAMs (CD11b^+^/Gr-1^−^/F4/80^+^) isolated from LLC tumors of Slc40a1^C326S^ mice, which express a ferroportin allele with a point mutation (C326S) that causes resistance to hepcidin-binding (31), did not express ferroportin protein in most TAMs (Figure 2D) pointing toward a hepcidin-independent downregulation of ferroportin. We conclude that iTAMs are hallmarked by a phenotype of hemoglobin recycling and iron retention.

### Hemorrhagic Areas in LLC Tumors Show Increased Inflammation

**Figure 3:**
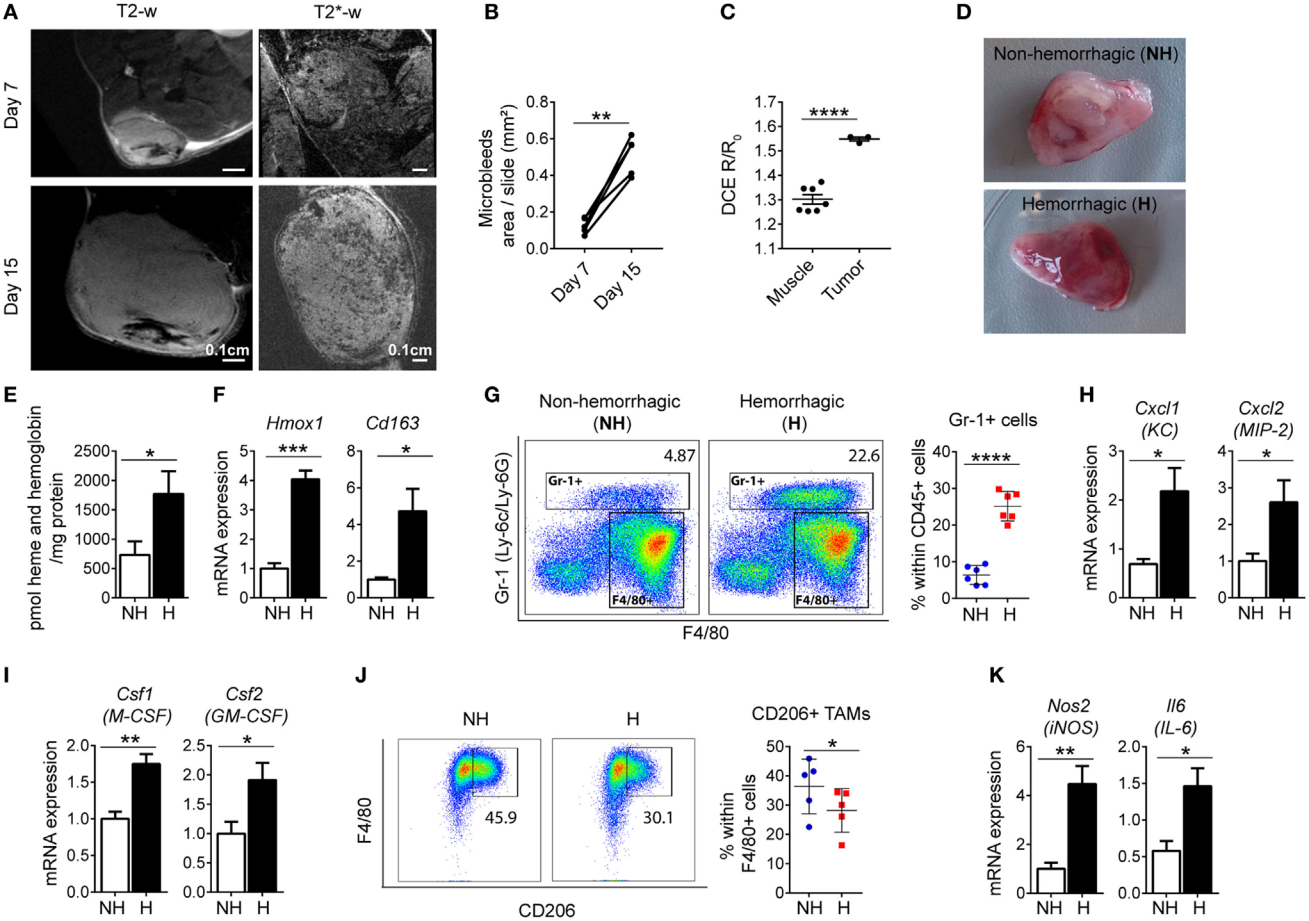
Hemorrhagic areas from Lewis lung carcinoma (LLC) tumors show increased inflammation. **(A)** Magnetic resonance imaging of LLC tumors, 7 and 15 days after LLC inoculation with T2-w sequence and T2*-w gradient echo sequence (representative of *n* = 8). **(B)** Quantification of micro-bleeds by T2*-w gradient echo sequence (*n* = 5). **(C)** Dynamic contrast-enhanced imaging in muscle (*n* = 7) and in tumor tissue (*n* = 3) at day 7 after LLC inoculation. **(D)** Co-existence of non-hemorrhagic (NH) area and hemorrhagic (H) area in a LLC tumor. **(E)** Heme and hemoglobin quantification in NH and H areas (*n* = 6). **(F)** *Hmox1* and *Cd163* mRNA expression determined by quantitative RT-PCR in total lysates of NH and H areas (*n* = 6). **(G)** Representative plots and quantification of Gr-1^+^ cells in NH and H areas (*n* = 6). **(H,I)** mRNA expression of *Cxcl1* and *Cxcl2*, **(H)** and *Csf1* and *Csf2* **(I)** determined by quantitative RT-PCR of total lysates of NH and H areas (*n* = 6). **(J)** Representative flow cytometry plots of F4/80^+^ CD206^+^ TAMs and quantification in NH and H areas (*n* = 5). **(K)** mRNA expression of *Nos2* and *Il6* determined by quantitative RT-PCR of total lysates of NH and H areas (*n* = 6). All mRNA levels were normalized to *Rpl19* mRNA expression and expressed as fold change relative to NH. All tumors were collected 15 days after LLC inoculation. Data are shown as mean ± SEM. *p < 0.05, **p < 0.01, ***p < 0.001, and ****p < 0.0001.

We observed that iTAMs co-localize with RBCs in the TME. We next quantified tumor micro-bleedings in response to LLC tumor growth by high field magnetic resonance imaging (MRI). Micro-bleedings were detectable at day 7 after LLC inoculation and increased significantly in number with tumor progression (Figures 3A,B). In addition, dynamic contrast-enhanced (DCE) imaging showed that tumor vessels were more permeable than those within muscle tissue (Figure 3C), and thus more fragile and leaky, leading to the occurrence of micro-bleedings within tumors. Heme, a product of RBC degradation, promotes inflammation by activating macrophages, neutrophils, and endothelial cells (23, 45). We next dissected hemorrhagic areas (H) and NH areas from the same tumor (Figure 3D) and analyzed their properties. Hemorrhagic areas showed increased heme levels and *Hmox1* mRNA expression (Figures 3E,F), as well as elevated *Cd163* levels (Figure 3F), consistent with the accumulation of iTAMs in areas of RBC extravasation. In addition, the percentage of Gr-1^+^ cells (gated as CD11b^+^/Gr-1^+^) was increased (Figure 3G). The marker Gr-1 (Ly-6C/Ly-6G) is expressed in neutrophils and granulocytes and myeloid-derived suppressor cells (35). Interestingly, expression levels of chemokines known for their neutrophil and myeloid cell chemoattractant activity, KC (*Cxcl1*) and MIP-2 (*Cxcl2*) were increased in hemorrhagic compared to NH areas (Figure 3H). In addition, expression of M-CSF (*Csf1*) and GM-CSF (*Csf2*) that drive macrophage differentiation were also elevated (Figure 3I). In hemorrhagic areas TAMs (CD11b^+^/Gr-1-/F4/80^+^) expressed less CD206, an M2 polarization marker, suggesting a shift of macrophages toward a pro-inflammatory phenotype (Figure 3J). Consistently, mRNA expression of the M1 markers *Nos2* and *Il-6* were increased in H areas (Figure 3K). In summary, we show that hemorrhagic areas occur due to RBC extravasation from permeable vessels in the TME and are characterized by an infiltration of leukocytes, the accumulation of CD206^low^ iTAMs, as well as increased inflammation.

### Hemolytic RBCs Induce Pro-inflammatory Responses in TAMs

**Figure 4:**
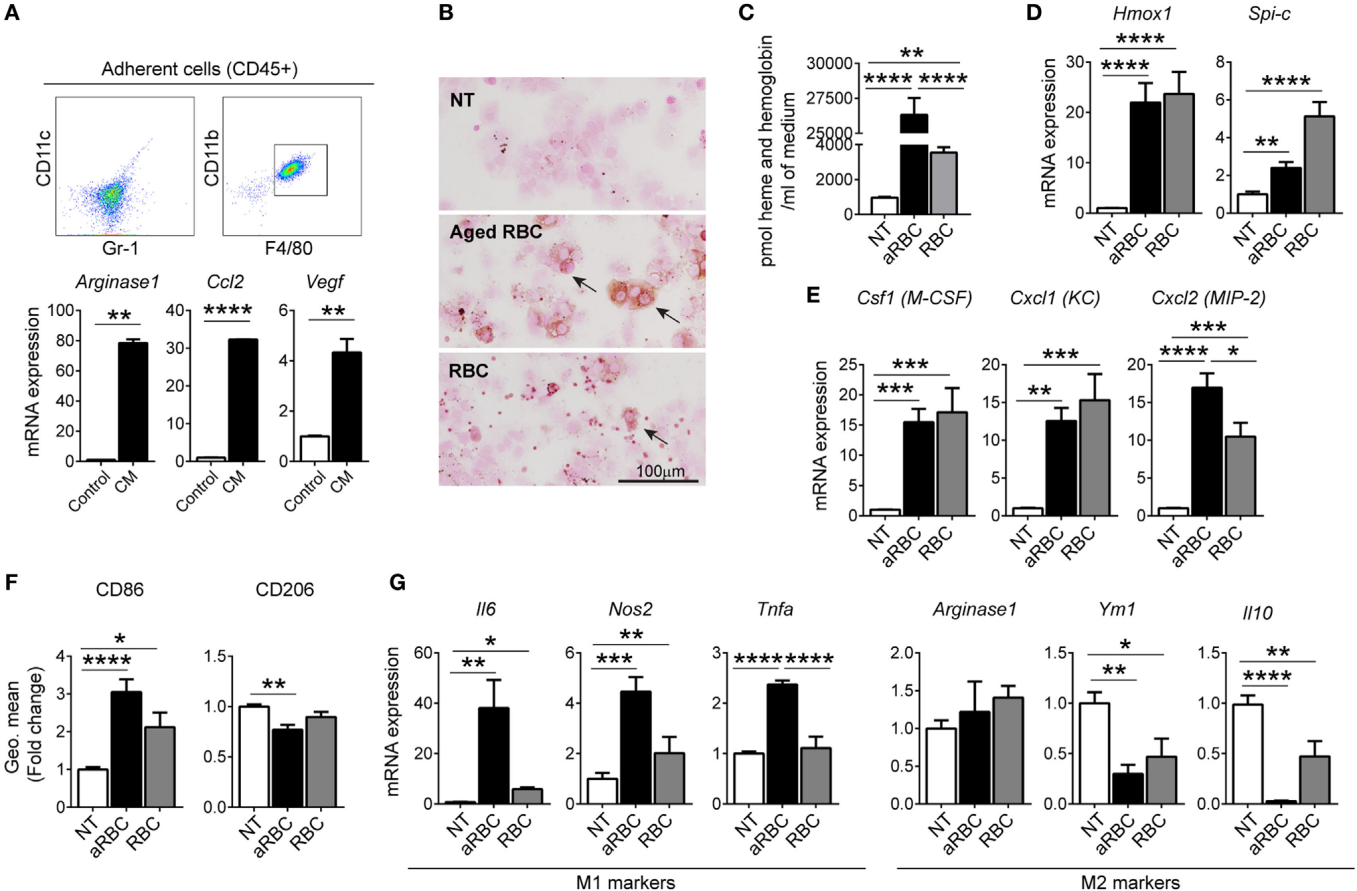
Hemolytic red blood cells (RBCs) shift tumor-associated macrophages (TAMs) polarization toward an M1-like phenotype. **(A)** Representative flow cytometry plots of BMDM differentiated with conditioned media (CM) from Lewis lung carcinoma (LLC) cells (*in vitro* TAMs) and mRNA expression of *Arginase 1, Ccl2*, and *Vegf* compared to control BMDM. **(B)** DAB enhanced Perls’ staining (indicated by arrows) of *in vitro* TAMs, non-treated (NT), treated with aged RBCs (aRBC) or RBCs (RBC) (representative of *n* = 3). **(C)** Heme and hemoglobin quantification in the supernatant of *in vitro* TAMs, NT, treated with aged RBCs (aRBC) or RBCs (RBC). **(D,E)** mRNA expression of *Hmox1* and *Spi-c* **(D)** and *Csf1, Cxcl1, and Cxcl2* **(E)** in *in vitro* TAMs, NT, treated with aged RBCs (aRBC) or RBCs (RBC), determined by quantitative RT-PCR. **(F)** Quantification of CD86 and CD206 expression by flow cytometry in *in vitro* TAMs NT, treated with aged RBCs (aRBC) or RBCs (RBC), results are shown as geometric mean fold change to NT samples (*n* = 9). **(G)** mRNA expression of M1 markers: *Il6, Nos2 and Tnfa and* M2 markers: *Arginase 1, Ym1, and Il10* in *in vitro* TAMs NT, treated with aged RBCs (aRBC) or RBCs (RBC). All cultures were analyzed 24 h after the respective treatment. All mRNA levels were determined by quantitative RT-PCR and normalized to *Rpl19* mRNA expression [shown as fold change to NT samples (*n* = 9)]. Data are shown as mean ± SEM. *p < 0.05, **p < 0.01, ***p < 0.001, and ****p < 0.0001.

To further understand the impact of RBC extravasation and degradation in TAMs, we next established an *in vitro* system by differentiating isolated bone marrow cells into TAMs. We incubated cells isolated from the bone marrow with (CM) from LLC cells for 4 days, and analyzed adherent cells that differentiated into macrophages (CD11c^neg^/Gr-1^neg^/CD11b^pos^/F4/80^pos^) (Figure 4A). We observed that macrophages differentiated with CM polarized toward an anti-inflammatory, tumor-tolerant phenotype showing increased expression of *Arginase 1, Ccl2*, and *Vegf* (Figure 4A). We next mimicked conditions in hemorrhagic areas of the TME by adding RBCs to macrophages. In order to mimic senescent hemolytic RBCs (e.g., as would be expected to occur in the prooxidant inflammatory TME) we prepared aged RBC by treating RBC overnight with calcium. *In vitro* TAMs accumulated iron when incubated with either aged RBCs (aRBC) (to mimic senescent/hemolytic RBCs) or non-aged RBCs (Figure 4B). Heme (heme and hemoglobin) concentration was significantly higher in the supernatant of macrophages treated with aged RBCs (Figure 4C). Additionally, heme was also detected in the supernatant on non-aged RBC, suggesting that inflammation may induce RBC breakage. Aged RBCs exposed to CM from LLC cells changed shape and size (higher FSC-A) and were more prone to hemolysis when compared to control media (Figure S4A–C in Supplementary Material). Furthermore, mRNA expression of *Hmox1* and *Spi-c*, markers for “iron recycling macrophages” was increased in macrophages treated with both RBCs sources (Figure 4D). Similar to observations in hemorrhagic areas of the tumor, the presence of RBCs increased mRNA expression of *Cxcl1* (KC), *Cxcl2* (MIP-2), and *Csf1* (M-CSF) (Figure 4E), suggesting that the activation of macrophages by heme/iron may trigger recruitment of myeloid cells. We further observed that in the presence of aged RBCs, pro-inflammatory markers (CD86, *Il6, Nos2*, and *Tnfa*) were increased, while the M2 markers remained unchanged (*Arginase 1*) or were decreased (CD206, *Ym1*, and *Il10*) (Figures 4F,G). Our results show that the responses of macrophages to RBCs in the *in vitro* model mirrored those in the hemorrhagic areas of the TME, with iron accumulation in macrophages (iTAMs) and a shift toward a pro-inflammatory phenotype.

### TAMs Exposed to Hemolytic RBCs Promote Tumor Cell Death

**Figure 5:**
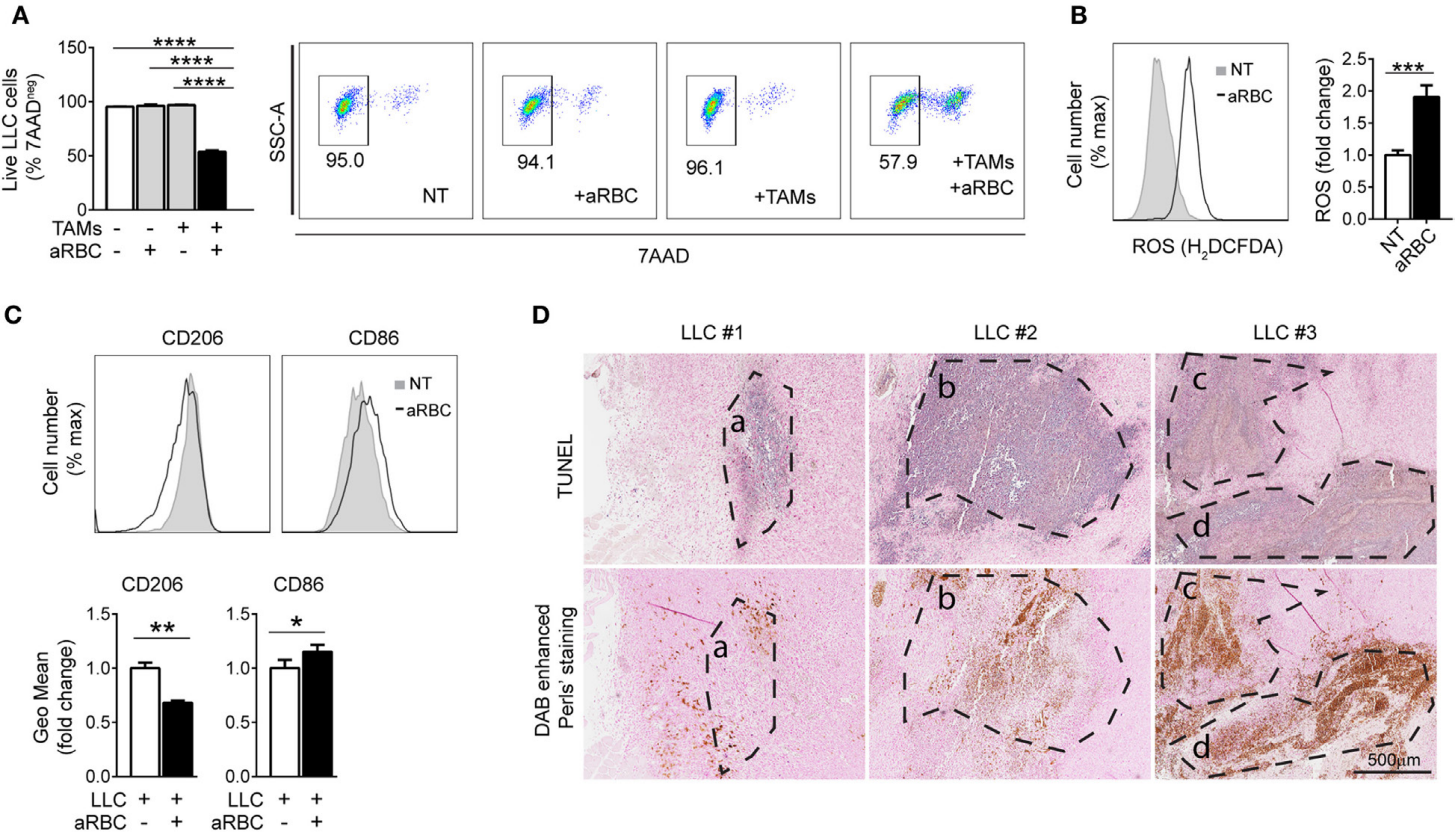
Macrophages exposed to hemolytic red blood cells (RBCs) promote tumor cell death. **(A)** Viability of Lewis lung carcinoma (LLC) cells co-cultured with *in vitro* tumor-associated macrophages (TAMs) and aged RBCs (aRBC) measured by flow cytometry. Results are shown as% of 7AAD negative cells (representative plots of LLC stained with 7AAD). **(B)** Representative plots and reactive oxygen species (ROS) quantification by flow cytometry in *in vitro* TAMs co-cultured with LLC cells and non-treated (NT) or treated with aged RBCs (aRBC). Results are shown as fold change to NT samples (*n* = 6). **(C)** Quantification of CD206 and CD86 expression by flow cytometry in *in vitro* TAMs co-cultured with LLC cells and aged RBCs (aRBC). Results are shown as geometric mean fold change to *in vitro* TAMs^+^LLC (white bar) (*n* = 9). All cultures were analyzed 24 h after the respective treatment. **(D)** Consecutive slides of LLC, showing four different areas overlapping **(A–D)** of tumors (upper panel) with TUNEL staining for apoptosis (purple staining) and (lower panel) DAB enhanced Perls’ staining (brown staining represents iTAMs and RBCs). Data are shown as mean ± SEM. *p < 0.05, **p < 0.01, ***p < 0.001, and ****p < 0.0001.

Pro-inflammatory macrophages in the TME can promote tumor cell death by producing ROS (46, 47). To test the effect of iron loading on tumor cell killing *in vitro*, we co-cultured macrophages exposed to hemolytic RBCs with LLC cells. This co-culture strongly reduced LLC cell viability (Figure 5A, gating strategy shown in Figure S2B in Supplementary Material); macrophages showed increased ROS levels (Figure 5B) and a shift toward pro-inflammatory phenotype characterized by the decreased expression of CD206 and increased expression of CD86 (Figure 5C). We next performed TUNEL staining and DAB enhanced Perls’ staining of consecutive slides of LLC tumors. Consistent with our findings in cultured cells, apoptotic areas co-localize with hemorrhagic areas and with the presence of iTAMs in the TME of LLC tumors (Figure 5D). Our findings demonstrate that macrophage iron loading in the TME correlates with and leads to a pro-inflammatory phenotype and anti-tumor activity. Thus, increasing the population of iTAMs in the TME emerged as a promising new therapeutical option to counteract tumor growth.

### Phagocytosis of Iron Nanoparticles by TAMs Inhibits Tumor Growth

**Figure 6:**
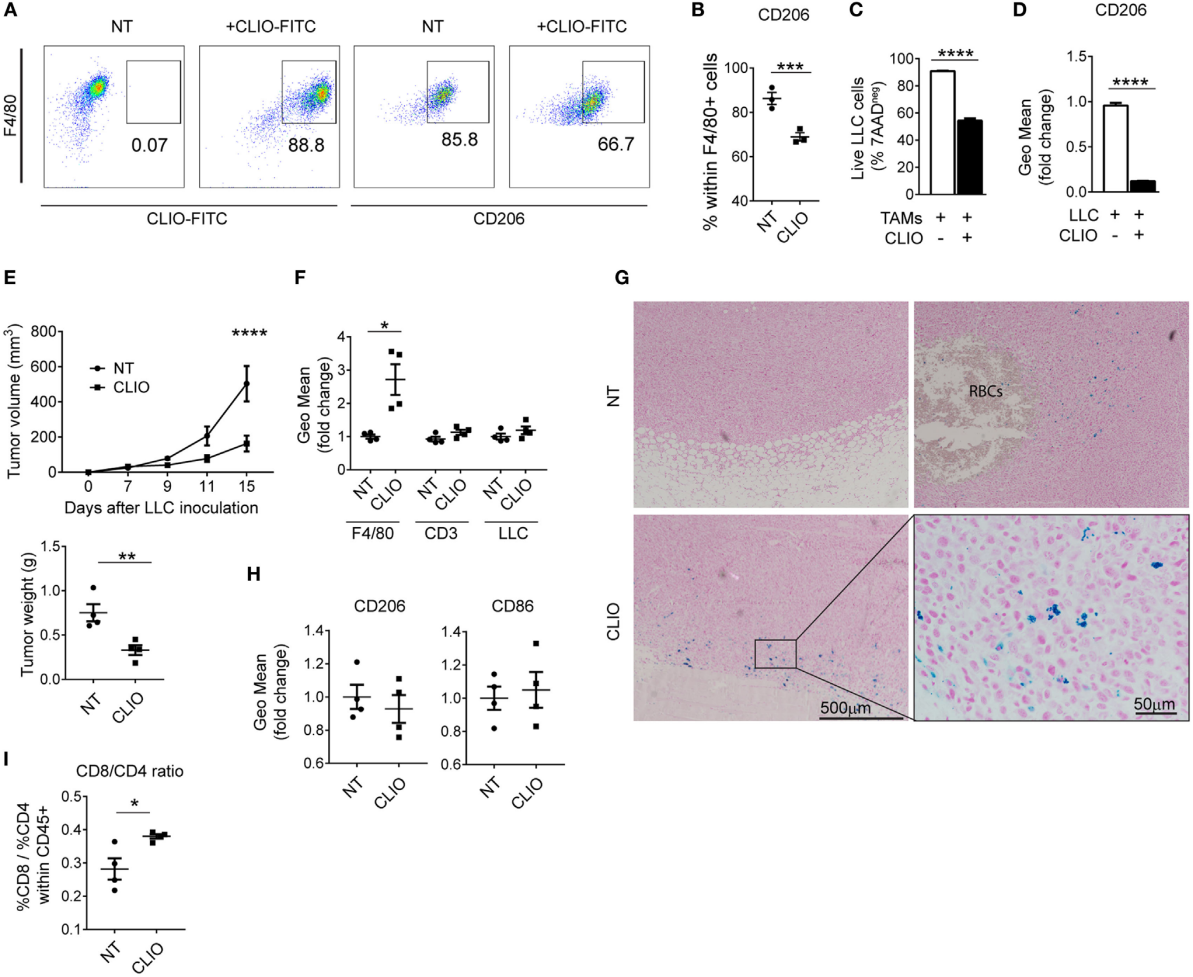
Iron nanoparticles accumulate in tumor-associated macrophages (TAMs) and delay tumor growth. **(A)** Representative plots of *in vitro* TAMs analyzed for the uptake of cross-linked iron oxide (CLIO)-FITC nanoparticles and expression of CD206. **(B)** Percentage of CD206 positive cells in *in vitro* TAMs (F4/80^+^), non-treated (NT) or incubated with CLIO-FITC nanoparticles. **(C)** Viability of Lewis lung carcinoma (LLC) cells measured by flow cytometry, in the presence of *in vitro* TAMs, CLIO-FITC at 48 h. Results are shown as% of 7AAD negative cells. **(D)** Expression of CD206 measured by flow cytometry in *in vitro* TAMs in the presence of LLC cells and incubated with CLIO-FITC, as geometric mean fold change (Geo Mean) to control (TAMs^+^LLC) at 48 h. **(E)** Tumor volume of sc LLC tumors NT or co-injected with CLIO-FITC nanoparticles and tumor weight at 15 days after LLC inoculation. **(F)** CLIO-FITC uptake in TAMs (F4/80), CD3^+^ and LLC cells measured by flow cytometry. Results are shown as geometric mean fold change (Geo Mean) compared to cells from NT tumors. **(G)** Representative Perls’ staining of LLC tumors NT or co-injected with CLIO-FITC nanoparticles (CLIO). RBCs are indicated in the NT sample. Blue staining represents iron-loaded TAMs. **(H)** Quantification of CD86 and CD206 expression by flow cytometry in F4/80^+^ cells in tumors NT or co-injected with CLIO-FITC nanoparticles (CLIO) results are shown as geometric mean fold change to NT samples (*n* = 9). **(I)** Ratio of the% of CD8/CD4 cells within CD45^+^ cells in tumors non-treated (NT) or co-injected with CLIO-FITC nanoparticles (CLIO). All tumors were collected 15 days after LLC inoculation. Data are shown as mean ± SEM. *p < 0.05, **p < 0.01, ***p < 0.001, and ****p < 0.0001.

We tested CLIO nanoparticles as a strategy to deliver iron to macrophages without provision of this growth factor to tumor cells. CLIO nanoparticles are used for *in vivo* imaging by magnetic resonance and are specifically ingested by phagocytic cells such as TAMs, rather than neighboring cell types such as tumor cells or other leukocytes (48). Similar to TAMs exposed to RBCs, macrophages accumulated CLIO nanoparticles (Figure 6A) which induced decreased expression of CD206 (Figures 6A,B). We next tested whether TAMs treated with CLIO displayed anti-tumor activity *in vivo* and *in vitro*. Incubation of LLC cells with CLIO-treated TAMs significantly reduced tumor cell viability (Figure 6C), correlating with a decrease in the expression of CD206 in TAMs (Figure 6D). We injected mice with LLC cells and analyzed tumor growth with or without co-injection of CLIO. 15 days after injection, tumors from CLIO-co-injected mice were significantly smaller than control tumors (Figure 6E). The CLIO nanoparticles accumulated in TAMs (Figure 6F), which were predominantly localized in the periphery of the tumor and near the invasive front (Figure 6G). The presence of iTAMs was also detected in NT samples, near areas of RBCs extravasation (Figure 6G). In accordance, we observed a moderate reduction of CD206 expression and increased CD86 expression (Figure 6H), together with a significant increase of the CD8/CD4 T-cell ratio (Figure 6I). These data show that the injection of iron nanoparticles limits tumor growth in the LLC cell mouse model, translating the *in vitro* effect observed with hemolytic RBCs. Our data suggest that iron nanoparticles injection could be developed as a therapeutic strategy to inhibit tumor growth via TAMs reprogramming.

## Discussion

**Figure 7:**
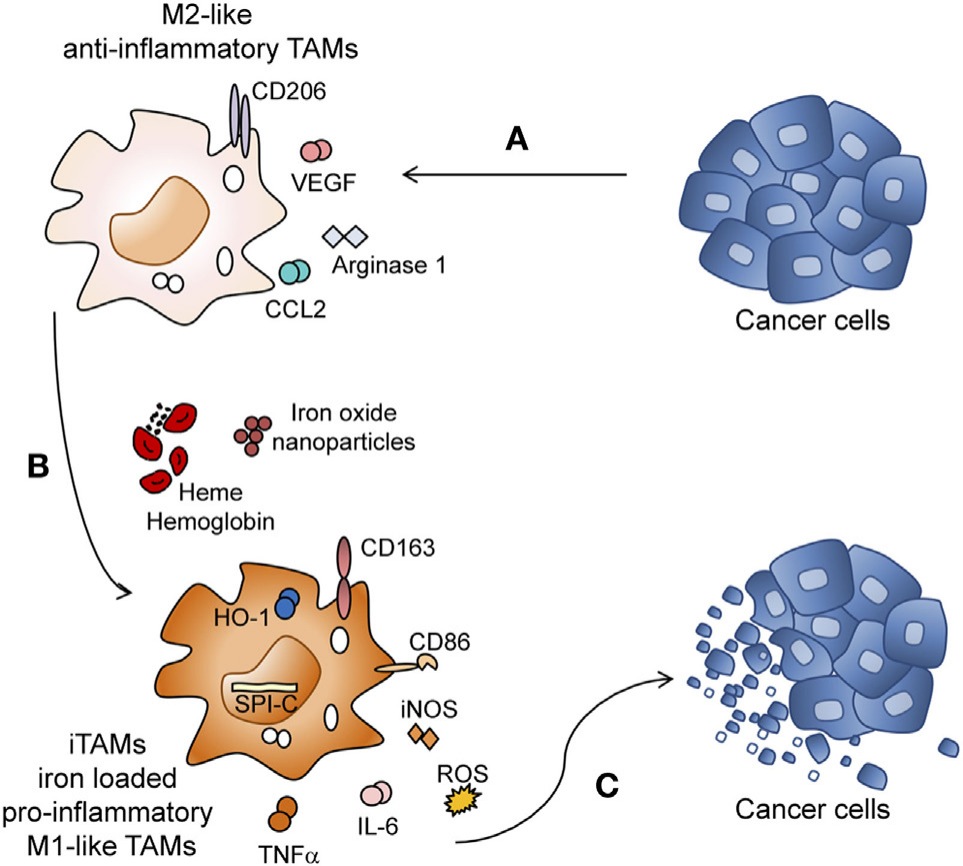
Heme and iron shift the polarization of tumor-associated macrophages (TAMs) toward a pro-inflammatory phenotype. **(A)** Lewis lung carcinoma (LLC) tumor cells promote M2 polarization of TAMs by inducing the expression of CD206, *Arginase 1, Ccl2*, and *Vegf*. **(B)** M2 macrophages exposed to the degradation products of hemolytic RBCs (heme, hemoglobin, and iron) or iron nanoparticles are reprogrammed to iron-loaded TAMs (iTAMs), with an M1-like inflammatory phenotype (increased production of *Tnfa, Il6* and reactive oxygen species (ROS) and increased expression of *Nos2* (iNOS), CD86, *Cd163, Spi-c*, and *Hmox1*). **(C)** iTAMs show tumoricidal activity by decreasing the viability of LLC cells.

In most malignancies, anti-inflammatory TAMs are detected in the TME which frequently correlates with poor prognosis (2, 49–53). Although TAMs could have the ability to eliminate tumor cells, they rather display a tumor supportive phenotype in most tumors, promoting angiogenesis and exerting immune suppressive functions (Figure 7A) (52). Thus, converting macrophages from a pro-tumoral to anti-tumoral phenotype is relevant for anti-cancer therapy. So far, iron was seen as an essential nutrient for tumor cell growth (54) but its contribution to immune responses in the TME remained unexplored.

Here, we discover a novel role of RBCs, heme and iron in the TME, which shapes the immune response. Hemolytic RBCs that extravasate from vessels during neoangiogenesis have the capacity to reprogram M2-like TAMs into pro-inflammatory (M1-like) TAMs with an ability to kill tumor cells. iTAMs show low expression of the iron exporter ferroportin, suggesting an inability to provide iron as a growth factor to the tumor. Of note, ferroportin expression is decreased at the mRNA level independent of hepcidin activity. iTAMs are further hallmarked by the expression of CD86^high^, CD206^low^, *Cd163*^high^, *Hmox1*^high^, show a pro-inflammatory phenotype associated with the production of ROS and pro-inflammatory cytokines (TNFα and IL-6), and are capable of killing tumor cells (Figures 7B–C). Interestingly, the same characteristics of this subset of macrophages can be elicited by applying exogenous iron sources, such as iron oxide nanoparticles.

Our findings in the TME are reminiscent of observations in hemolytic disease where heme and iron polarize splenic and liver macrophages toward an M1-like phenotype, contributing to inflammation and tissues damage (23). In accordance with our findings, a pro-inflammatory response of macrophages to heme and iron was further described in the wound-healing process, in the injured spinal cord and in hemophilic mice (24, 25, 55). Furthermore, free heme sensitizes hepatocytes to TNF-α and oxidative stress-induced cell death (56). Here, we show that hemolytic RBCs do not directly promote tumor cell death but require the M1-like pro-inflammatory activation of TAMs, which release pro-inflammatory cytokines and ROS (Figures 5A,B).

Non-small cell lung cancer patients with detectable iron in the TME have significant smaller tumors (Figure 1F). This supports the idea that exposure of TAMs to hemolytic RBCs and subsequent iron retention, which promotes their inflammatory phenotype, impacts on tumor cell viability (28, 57). In fact, the presence of M1 macrophages in several tumor entities such as NSCLC has been associated with better patient survival (58–61). By contrast, in breast cancer, TAMs are characterized by an “iron-donor” phenotype and ferroportin expression is detectable (62). These differences in the TAM phenotypes are likely explained by the cytokine/chemokine composition of the tumor niche. Key players may be M-CSF (*Csf1*) and GM-CSF (*Csf2*), which are implicated in the control of ferroportin expression (63, 64). GM-CSF suppresses ferroportin mRNA expression (64), thus the ratio of GM-CSF/M-CSF in the tumor tissue may explain ferroportin expression levels in macrophages. The composition of the TME, including the presence of RBCs directly affects macrophage polarization as well as the expression of iron genes. We studied lung cancer as a model, but we believe that this concept can be further extended to other tumor entities. Future studies will aim to understand how RBCs in the TME of other tumor subtypes affects TAM function. This knowledge will be important to design anti-tumor therapies.

Dependent on the pathophysiological context iron-induced M1 pro-inflammatory macrophage polarization can be detrimental or beneficial, thus representing an interesting target for therapy. In hemolytic disorders, heme and iron-dependent M1-like macrophage polarization contributes to inflammation and tissue damage, an effect which can be prevented by applying heme and iron scavengers (23). In the context of the TME, iron-mediated reprogramming of pro-tumoral M2-like TAMs into anti-tumoral M1-like TAMs is desirable to prevent tumor growth. We expect that this knowledge can be applied therapeutically by delivering iron to TAMs.

The “natural” population of iTAMs appears only in late stages of tumor development, when the angiogenic switch occurs and tumors are already well established. In this scenario, the number of iTAMs may be insufficient to exert a sufficiently large, protective anti-tumor response. Nevertheless, we observed that tumors with iTAMs are of significantly smaller size (Figure 1F), suggesting that iTAMs may impact on tumor growth. Our observations motivated us to use iron nanoparticles as a “specific” means to deliver iron to TAMs in early stages of tumor development. Application of iron nanoparticles to diminish tumor growth served as a proof-of-concept for the therapeutic potential of such an approach *in vitro* and in a mouse tumor model. Recently, ferumoxytol, another type of iron nanoparticle, was shown to reduce growth of subcutaneous adenocarcinoma and to prevent the development of liver metastasis by promoting a pro-inflammatory type of macrophages (65). We propose that the iron moiety of the nanoparticle plays a key role in determining TAM polarization and anti-tumoral activity. We suggest that the administration of iron formulations at early stages of tumor development will contribute to the inhibition of tumor growth, representing as a promising strategy for cancer therapy. Increasing the physiological population of TAMs via iron/heme delivery is expected to produce only mild adverse effects for cancer patients. However, the development of “iron sources” for targeted delivery to the TME is required. We expect that such a therapeutic approach will be of benefit in combination with current therapies, such as immunotherapy, to improve anti-cancer responses. Immune check point inhibitors, such as drugs that block programmed cell death-1 (PD-1) were shown to activate the immune system and trigger anti-tumor activity. These drugs are currently being applied to treat NSCLC (66, 67). PD-1 is expressed in the surface of macrophages and binds to the receptor PD-L1 on the surface of T cells, reducing cytokine production and suppressing T-cell proliferation (68). As a combination approach, increasing the population of iTAMs, together with the use of monoclonal antibodies or drugs that block T-cell inhibition, is expected to improve the immune response against cancer. T-cell activation requires the interaction of CD28 expressed on the T-cell surface with CD80 or CD86 expressed by macrophages (69). Since iTAMs express high levels of CD86, the amplification of the iTAM population may boost T-cell activation and suppress tumor growth. Consistently, we observe an increase of the CD8 cytotoxic T cells/CD4 helper T cells ratio in tumors treated with iron nanoparticles (Figure 6I).

In conclusion, we defined a novel pro-inflammatory niche within the TME. This niche contains hemorrhagic areas, where RBCs release heme and iron which is subsequently taken up by TAMs. Heme and iron differentiate M2-like TAMs into a M1-proinflammatory phenotype capable of reducing tumor growth. We further provide a proof-of-concept that iron nanoparticle treatment of TAMs reduces tumor growth. Our findings have potential to be further explored for translation into clinical applications to improve cancer therapy.

## Ethics Statement

Paraffin slides were provided by the Lung Biobank Heidelberg, a member of the Biomaterial bank Heidelberg (BMBH) and the Biobank platform of the German Center for Lung Research (DZL). Tissue microarrays (TMAs) were provided by the tissue bank of the National Center for Tumor Diseases (NCT, Heidelberg, Germany), in accordance with the regulations of the tissue bank and the approval of the ethics committee of Heidelberg University. Animal Experiments were approved by “Regierungspräsidium Karlsruhe” Germany, under the project number G267/12.

## Author Contributions

MCS designed the project, performed the experiments and wrote the manuscript; MOB performed the MRI experiments and gave advice on nanoparticle experiments; FV provided advice for experiments, critical discussion and wrote the manuscript; MPC, AS and CMT helped with some experiments; MM, TM and AW provided human histology slides and patient data; MP provided critical reading and discussion; MWH provided critical reading and wrote the manuscript; AC and MUM designed and supervised the project and wrote the manuscript.

## Conflict of Interest Statement

The authors declare that the research was conducted in the absence of any commercial or financial relationships that could be construed as a potential conflict of interest.
