# Supplementary material for: Iron Induces Anti-tumor Activity in Tumor-Associated Macrophages
Source: Front Immunol. 2017 Nov 8;8:1479. doi: 10.3389/fimmu.2017.01479 (PMC5682327; doi:10.3389/fimmu.2017.01479)
Supplement: Supplementary file 1 [file Data_Sheet_1.PDF]

SUPPLEMENTARY INFORMATION

Iron induces anti-tumor activity in tumor-associated macrophages

Milene Costa da Silva[1-5], Michael O. Breckwoldt[6,7], Francesca Vinchi[1,2], Margareta P. Correia[4], Ana Stojanovic[4], Carl Maximilian Thielmann[1,2], Michael Meister[5,8,], Thomas Muley[5,8,], Arne Warth[5,9], Michael Platten[6,7], Matthias W. Hentze[2], Adelheid Cerwenka[4,10†], Martina U. Muckenthaler[1,2,5,†]*

SUPPLEMENTARY MATERIAL AND METHODS

Cell culture

LLC cells were grown at 37°C in a humidified atmosphere of 5% CO_2/95% air in DMEM medium supplemented with 10% heat-inactivated fetal bovine serum and 1% penicillin/streptomycin (Sigma) under sterile tissue culture conditions. To collect conditioned media (CM), LLC cells were plated at $1x10^4$/ml of complete DMEM (10%FBS, 1%PenStrep) and CM was collected when LLC cells were about 80% confluent. CM was passed through a 0.22 µm filter and stored at -20°C until further use.

Flow cytometry and measurement of intracellular ROS

Cells were resuspended in 100 µl supernatant from 2.42G cells and placed at 4°C for 15 mins to block non-specific FC receptor binding. Cells were then washed with FACS buffer (PBS, 1% FBS and 0.1% sodium azide), incubated with fluorescently labeled antibodies (antibodies used are listed in Table S1) at 4°C in the dark for 30 mins followed by further washing with FACS buffer. For ROS quantification, macrophages were incubated with 5 µM H_2DCFDA (Molecular Probes, Inc., Eugene, OR) in Hanks' balanced salt solution (HBSS) for 30 mins at 37 °C under 5% CO_2 atmosphere. Cells were resuspended in FACS buffer, and evaluated or

sorted using a FACSAria II (Becton Dickinson) flow cytometer. Cell death was determined using 7AAD (BioLegend). The expression of surface markers and ROS quantification is shown as Geometric mean (Geo Mean) compared to the control condition. The geometric mean fluorescence intensity (MFI) of the cells stained with the isotype-matched antibody or unstained samples (auto-fluorescence for ROS quantification) was subtracted from the MFI of those stained with the specific antibody or for ROS. Data were further analyzed using the FlowJo software (Tree Star).

Co-culture of *in vitro* TAMs with RBC and LLC cells

LLC cells (10^5/ml) were added to *in vitro* TAMs (after 4 days of differentiation in LLC CM) or kept in CM (same as the one used to differentiate *in vitro* TAMs) for 12h. Afterwards, cells were treated as indicated: non-treated (NT), RBC (10^8/ml) or aged RBC (aRBC) (10^8/ml), for 24h. All cells were removed and labelled for flow cytometry analysis (gating strategy in Fig. S2B), or used for cytospin preparation and RNA extraction. Supernatant of the co-cultures was collected and kept at -80°C until further analysis.

Preparation of splenocytes for cytospin slides

Spleens were removed and passed through a 70 μm cell strainer. Red blood cell lysis was performed with ACK lysing buffer (Gibco, Life Technologies). Cells were washed in PBS, 1500 r.p.m. for 10 mins and resuspended in 2mls of PBS and used for cytospin preparations.

Magnetic resonance imaging

MRI was performed on a 9.4-Tesla (T) horizontal-bore small animal MRI scanner (BioSpec 94/20 USR; Bruker BioSpin GmbH) with a spine phased-array surface receiver coil. The MRI protocol included a standard T2-weighted (T2-w) rapid acquisition with relaxation enhancement (RARE) sequence to assess tumor anatomy. A T2*-w FLASH sequence was used to assess microbleedings (hypointense signals within the tumor): 3D sequence: 80 μm isotropic resolution; TE: 18 ms; TR: 50 ms; flip angle: 12°; number of averages: 1; acquisition matrix: 400 × 188 × 100; duration: 15 min 40 s. Dynamic contrast-enhanced (DCE) imaging was used to assess vascular permeability (TE: 1.8 ms; TR: 16 ms; flip angle:

10°; slice thickness: 700 mm, acquisition matrix: 66 x 128, 3 slices acquired, number of averages: 1, 300 repetitions; 700 mm in plane resolution; duration: 10 min, time resolution 2 s). 0.2 mmol/kg Gadodiamide (Omniscan, Nycomed, Ismaningen, Germany) was administered by tail vein injection during the DCE imaging. MR images were visualized in OsiriX Imaging software (version 4.12; Pixmeo). For the quantification of microbleedings, T2* hypointense areas were segmented semiautomatically in AMIRA (FEI). Quantification of DCE images was performed in FIJI. ROIs were placed in the tumor and adjacent muscle. Intensity values were measured and signal ratios R/R_0, were calculated (signal after Gd-contrast injection / before contrast injection).

RNA isolation and qRT-PCR analysis

Total tissue RNA was isolated using TRIzol (Life Technologies). RNA from cells was extracted using the RNeasy Mini Kit (Qiagen) or Arcturus Picopure RNA Isolation Kit, (Applied Biosystems). 0.5 µg to 1 µg of total RNA was reverse transcribed by using RevertAid H Minus reverse transcriptase (Thermo Scientific), random primers (Invitrogen) and dNTPs (Thermo Scientific). Quantitative RT-PCR was performed using SYBR green on a Step One Plus Real Time PCR System (Applied Biosystems, California, USA). The primers used are listed in Table S2. Differences in relative quantification are shown as fold-change compared to the control condition. Ribosomal protein L19 (RPL19) was used to normalize cDNA levels.

Protein quantification, heme and hemoglobin measurements

Protein lysates were obtained by homogenizing snap-frozen tissues or cell suspension in RIPA buffer supplemented with protease inhibitors (Roche). Protein concentration was determined using the DC protein assay (Bio-Rad). Protein samples (10 µg) or cell culture supernatant (25 µL) were incubated with 0.5 ml of 2 M Oxalic Acid (Sigma-Aldrich) at 95°C for 30 mins and centrifuged (10000 r.p.m., 5 mins). Fluorescence emission in the supernatant was determined in a spectrofluorimeter (SpectraMax, Molecular Devices). Excitation and emission wavelengths were set at 405 and 662 nm, respectively. The background was evaluated by measuring fluorescence in non-boiled samples.

Measurement of cytokines

Cytokine protein levels were determined in the serum of mice applying Multiplex bead-array based technology (BioPlex200 System) using the Bio-Plex Pro Cytokine Reagent Kit and Bio-Plex Pro Mouse Cytokine sets (Bio-Rad) according to manufacturer's instructions.

SUPPLEMENTARY FIGURES

FIGURE S1

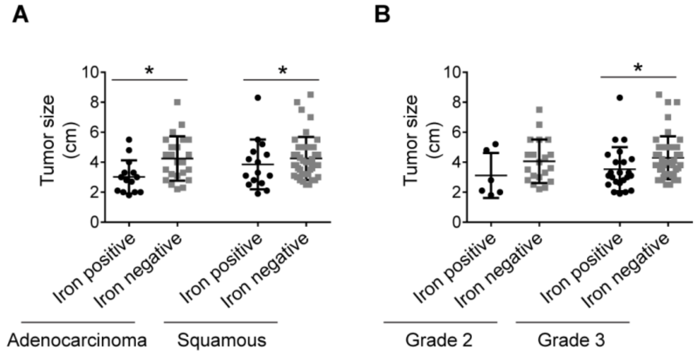

Figure S1. NSCLC tumors positive for iron staining in TAMs show a significantly smaller size. (A) Comparison of tumor size in a cohort of NSCLC patients separated by histological subtype and iron content: adenocarcinoma iron positive (n=14); adenocarcinoma iron negative (n=22); squamous carcinoma iron positive (n=15) and squamous carcinoma iron negative (n=43). **(B)** Comparison of tumor size in a cohort of NSCLC patients divided by grade and iron content: grade 2 iron positive (n=6); grade 2 iron negative (n=20); grade 3 iron positive (n=23) and grade 3 iron negative (n=45). Data are shown as mean ± SEM. *$p < 0.05$, **$p < 0.01$, ***$p < 0.001$, and ****$p < 0.0001$.

FIGURE S2

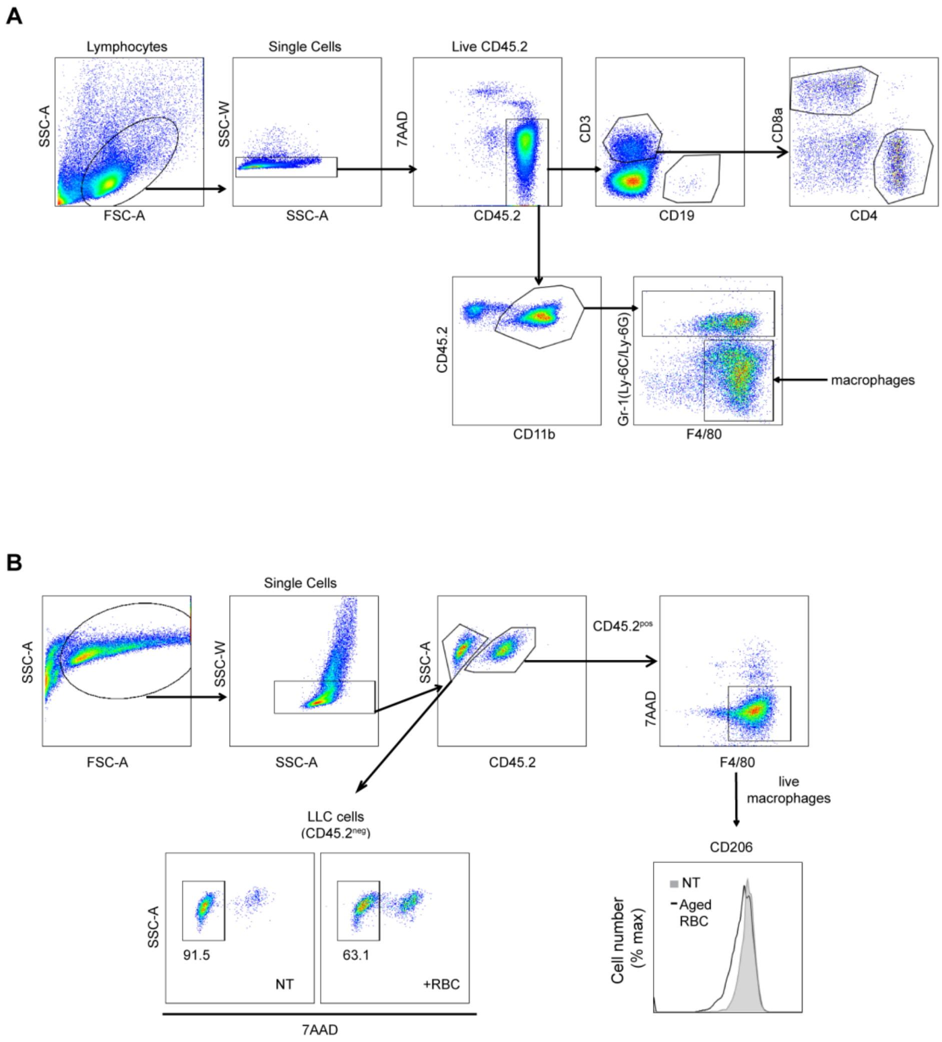

Figure S2. Gating strategy applied to analyze tumor cell suspensions and *in vitro* co-cultures. (A) Gating strategy to select macrophages by flow cytometry, from tumor infiltrates. After selection of single live CD45.2 cells, the analysis of polarization markers was performed within CD11b+/Gr-1-/F4/80+ cells. **(B)** Gating strategy to analyze co-cultures of *in vitro* TAMs and LLC cells. Cells were separated in $CD45^{neg}$ (LLC cells) and $CD45^{pos}$. Within the $CD45^{neg}$ population, cells were analyzed for viability by 7AAD quantification. Examples are shown of the percentage of live LLC cells in co-culture with *in vitro* TAMs alone (NT) or in the the presence of RBC (+RBC). Within the $CD45^{pos}$ cells, F4/80 positive/7AAD negative

cells (live TAMs) were analyzed for the expression of polarization markers (CD206 shown as an example) and ROS.

FIGURE S3

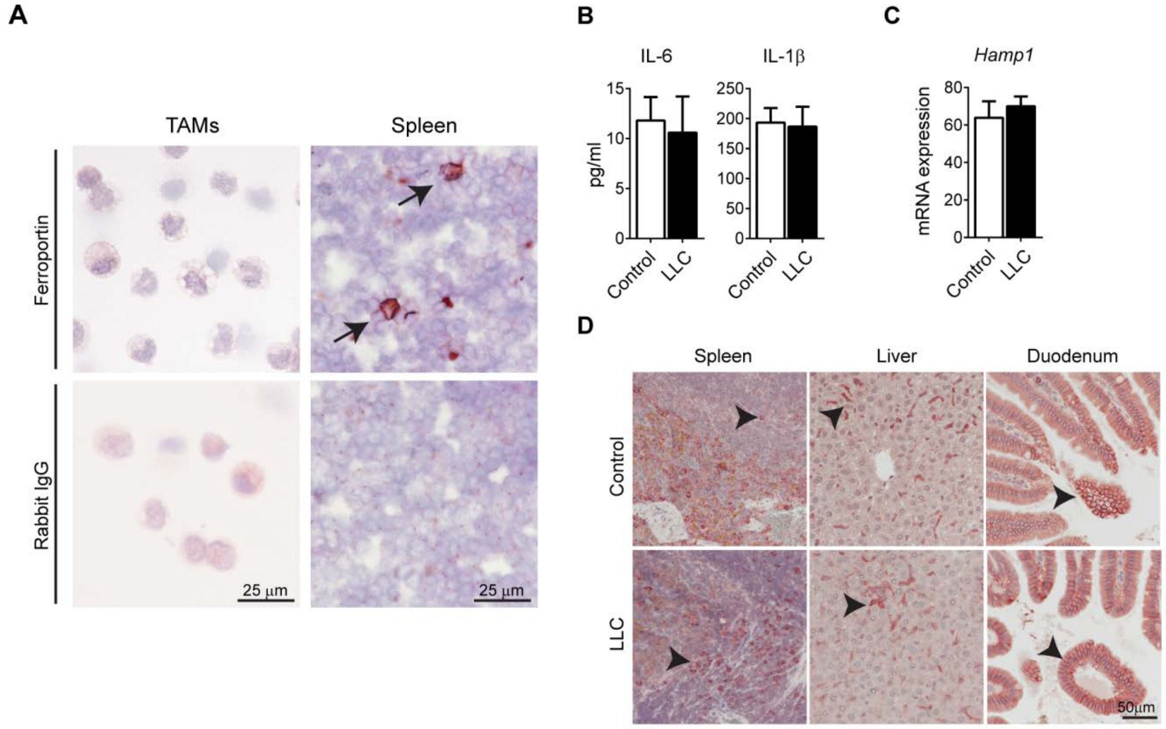

Figure S3. Hepcidin mRNA levels in tumor-bearing mice remain unaltered. (A) Anti-ferroportin staining and IgG isotype control in TAMs and splenocytes, (representative of 4 mice) Arrows indicate splenic macrophages positive for ferroportin staining. **(B)** IL-6 and IL-1β levels in serum of non-injected mice (control, n=5) and LLC-bearing mice (LLC, n=8) shown as pg/ml of serum. **(C)** *Hamp1* mRNA relative expression in liver from control (n=8) and LLC-bearing mice (n=14) determined by quantitative RT-PCR. **(D)** Anti-ferroportin staining (indicated by arrows) in spleen, liver and duodenum from control and LLC-bearing mice (representative of 4 mice). mRNA levels were normalized to *Rpl19* mRNA expression and all tissues were collected 15 days after LLC inoculation. Data are shown as mean ± SEM. *$p < 0.05$, **$p < 0.01$, ***$p < 0.001$, and ****$p < 0.0001$.

FIGURE S4

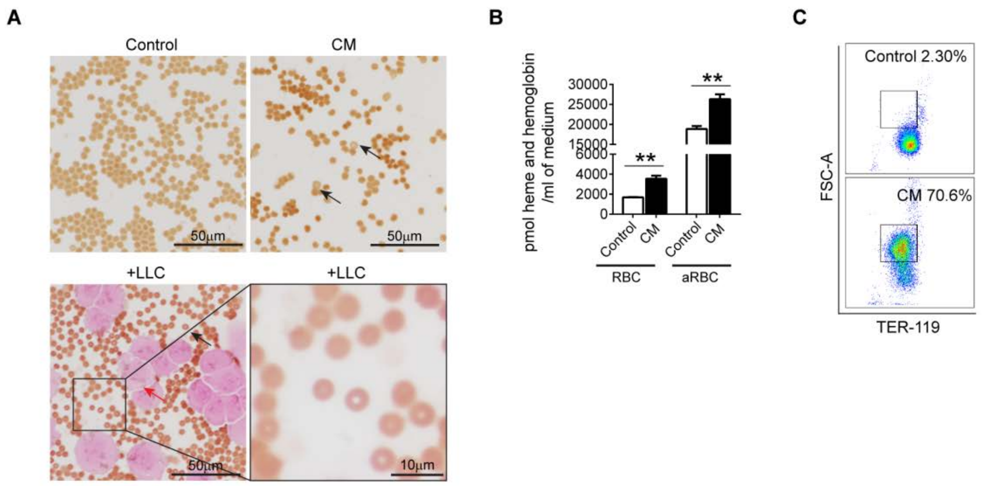

Figure S4. Red blood cells change shape in the presence of CM from LLC cells. (A) DAB enhanced Perls' staining of RBC cultured in DMEM (control), CM of LLC cells or in co-culture with LLC cells. Black arrows point to deformed RBC and the red arrow points to LLC cells. **(B)** Heme and hemoglobin quantification in the supernatant of RBC and aged RBC incubated with control media (DMEM) or CM of LLC cells. **(C)** Flow cytometry plots of RBC incubated with control media (DMEM) or CM of LLC cells. All cultures were analyzed 24h after the respective treatment. Data are shown as mean ± SEM. $*p < 0.05$, $**p < 0.01$, $***p < 0.001$, and $****p < 0.0001$.

SUPPLEMENTARY TABLES

Table S1. Antibodies used for Flow cytometry (anti-mouse)

Antibody	Fluorophore	Clone	Isotype	Manufacturer
**F4/80**	APC	BM8	Rat IgG2a, κ	BioLegend
**CD11b**	APC-Cy7	M1/70	Rat IgG2b, κ	BD Pharmingen
	FITC	M1/70		BDPharmingen
	Horizon V500	M1/70		BD Horizon
**GR1**	Alexa Fluor 700	RB6-8C5	Rat IgG2b, κ	BioLegend
**CD3**	FITC	17A2	Rat IgG2b, κ	BioLegend
**CD19**	PE	6D5	Rat IgG2a, κ	BioLegend
**CD4**	PE-Cy7	RM4-5	Rat IgG2a, κ	BioLegend
**CD8a**	APC	53-6.7	Rat IgG2a, κ	BioLegend
**CD206**	FITC	MR5D3	Rat IgG2a, κ	BioLegend
	Alexa Fluor 700	MR5D3	Rat IgG2a, κ	BioLegend
**CD86**	PE	GL-1	Rat IgG2a, κ	BioLegend
**CD45.2**	Pacific Blue	104	Mouse (SJL) IgG2a, κ	BioLegend
**Ter119**	Alexa Fluor 700	TER-119	Rat IgG2b, κ	BioLegend

Table S2. Primers for quantitative RT-PCR (*mus musculus*)

Gene	Sequence
Rpl19	Forward 5' AGGCATATGGGCATAGGGAAGAG 3'<br>Reverse 5' TTGACCTTCAGGTACAGGCTGTG 3'
Hamp1	Forward 5'CCTATCTCCATCAACAGAT 3'<br>Reverse 5'TGCAACAGATACCACACTG 3'
Cd163	Forward 5’ TCTCAGTGCCTCTGCTGTCA 3’<br>Reverse 5’ CGCCAGTCTCAGTTCCTTCT 3’
Hmox1	Forward 5' AGGCTAAGACCGCCTTCCT 3'<br>Reverse 5' TGTGTTCCTCTGTCAGCATCA 3'
Fpn	Forward 5' TGTCAGCCTGCTGTTTGCAGGA 3'<br>Reverse 5' TCTTGCAGCAACTGTGTCACCG 3'
Arginase 1	Forward 5' AATCTGCATGGGCAACCTGT 3'<br>Reverse 5' GTCTACGTCTCGCAAGCCAA 3'
Ccl2	Forward 5' CATCCACGTGTTGGCTCA 3'<br>Reverse 5' GATCATCTTGCTGGTGAATGAGT 3'
Cxcl1	Forward 5’AGACTCCAGCCACACTCCAA 3’<br>Reverse 5’ TGACAGCGCAGCTCATTG 3’
Cxcl2	Forward 5’AAAATCATCCAAAAGATACTGAACAA 3’<br>Reverse 5’CTTTGGTTCTTCCGTTGAGG 3’
Csf1	Forward 5' GGTGGAACTGCCAGTATAGAAAG 3'<br>Reverse 5' TCCCATATGTCTCCTTCCATAAA 3'
Csf2	Forward 5' GCATGTAGAGGCCATCAAAGA 3'<br>Reverse 5' CGGGTCTGCACACATGTTA 3'
Nos2	Forward 5' TGGAGACTGTCCCAGCAATG 3'<br>Reverse 5' CAAGGCCAAACACAGCATACC 3'
Tnfa	Forward 5' TGCCTATGTCTCAGCCTCTTC 3'<br>Reverse 5' GAGGCCATTTGGGAACTTCT 3'
Ym1	Forward 5' CCAGCAGAAGCTCTCCAGAAGCA 3'<br>Reverse 5' GGCCTGTCCTTAGCCCAACTGGT 3'
Il-6	Forward 5' GCTACCAAACTGGATATAATCAGGA 3'<br>Reverse 5' CCAGGTAGCTATGGTACTCCAGAA3'
Tfr1	Forward 5' CCCATGACGTTGAATTGAACCT 3'<br>Reverse 5' GTAGTCTCCACGAGCGGAATA 3'
Il-10	Forward 5' ACTGCACCCACTTCCCAGT 3'<br>Reverse 5' GTCCAGCTGGTCCTTTGTTT 3'
Spi-c	Forward 5' TCCGCAACCCAAGACTCTTCAA 3'<br>Reverse 5' GGGTTCTCTGTGGGTGACATTCCAT 3'
Vegf	Forward 5' CCCACTGAGGAGTCCAACATC 3'<br>Reverse 5' GGCCTTGGTGAGGTTTGATC 3'
